# The Frequency of *qnr* Genes in Extended-Spectrum β-lactamases and non-ESBLs *Klebsiella pneumoniae *Species Isolated from Patients in Mashhad, Iran

**Published:** 2017-10-01

**Authors:** Nafiseh Izadi, Mahboubeh Naderi Nasab, Elnaz Harifi Mood, Zahra Meshkat

**Affiliations:** 1 *Student Research Committee (SRC), Mashhad University of Medical Sciences, Mashhad, Iran.*; 2 *Antimicrobial Resistance Research Center, Mashhad University of Medical Sciences, Mashhad, Iran* *.*

**Keywords:** *Klebsiella pneumonia*, Quinolones, Extended-Spectrum β-lactamases, Double Disk Synergy Test

## Abstract

**Background and Objectives:**

Since the fluoroquinolones are the broad-spectrum antibiotics, they affect both Gram-negative and Gram-positive bacteria. These antibiotics are widely prescribed by physicians. As a result, some bacteria, especially Enterobacteriaceae, have shown a resistance to this family of antibiotics**.** The current study aimed at detecting the frequency of *qnrA*, *qnrB, *and *qnrS *genes, novel plasmid-mediated quinolone-resistance genes, among extended-spectrum β-lactamases (ESBL)-positive and ESBL-negative* Klebsiella pneumoniae* isolates.

**Materials and Methods:**

One hundred and thirty isolates of *K. pneumoniae* were collected from Imam Reza Hospital and its associated clinics from May 2011 to July 2012. The isolates were tested for ESBLs by the conventional methods. Polymerase chain reaction (PCR) was performed to amplify *qnr A, B*, and *S*.

**Results:**

Thirty-eight (29.3%) isolates were ciprofloxacin-resistant. Among 130 *K. pneumoniae* infectious isolates, 56 (43%) were capable of producing ESBL; 10.8% (n=14), 15.4% (n=20), and 20.8% (n=27) of ESBL-producing *K. pneumonia* were positive for *qnrA, qnrS*, and *qnrB*, respectively, and 13.8% (n=18) of the isolates harbored 2 or 3 *qnr* genes.

**Conclusion:**

The results of the current study showed that quinolone-resistance genes were more frequent in ESBL-producing *K. pneumoniae* (37.5%) isolates, compared with the ESBL-negative isolates (20.89%). The prevalence of *qnr* genes was high in *K. pneumoniae* isolates, with higher frequency in ESBL-positive strains. Most of the isolates were positive for all 3 groups of *qnr* genes and the *qnrB* was the most common one.

## Introduction

Fluoroquinolones are the broad-spectrum antibiotics and affect both Gram-negative and Gram-positive bacteria. Their widely administration to treat bacterial infections led to increase in antibiotic resistance, especially among Enterobacteriaceae. Three mechanisms of resistance to quinolones are as follows: 1) Chromosomal mutations that modify the activity of DNA gyrase and DNA topoisomerase IV; 2) Overexpression of efflux pumps, which in turn decreases drug accumulation; and 3) Plasmids that carry *qnr* genes and are transferable from one bacterium to another (1-5).

 The *qnr* genes produce Qnr proteins (Qnr A, B, and S), which protect DNA gyrase from quinolone inhibition. The plasmids may also carry the genes encoding extended-spectrum β-lactamases (ESBLs) such as *SHV, TEM, CTX-M, KPC-2, aac(6')-Ib-cr* and *AmpC* genes (6-10). ESBLs confer resistance to a wide variety of cephalosporins (one of the most widely used drugs to treat bacterial infections). This resistance is brought by β-lactamase enzymes produced by a variety of *bla* genes such as *SHV, TEM, CTX-M, KPC-2, aac(6')-Ib-cr* and *AmpC* and the presence of *qnr* genes in the strains producing ESBLs. Firstly, plasmid-mediated quinolone-resistance was reported in the clinical isolates of *Klebsiella pneumoniae* (*K. pneumoniae*) from the USA in 1998(11). *K. pneumoniae* is capable of producing ESBLs and is a common cause of nosocomial infections. 

The coexistence of these 2 groups of genes (*qnr* and *bla*) in these bacteria is an issue for physicians or health care workers in hospitals. The spread of these strains increases the mortality, especially in the elderly and infants with compromised immune responses (12, 13). 

The high prevalence of *qnr* genes were reported in different regions of the world (14). The prevalence of *qnr* genes in *K. pneumoniae* species was reported 48.9% in Malaysia (15), 40.5% in Korea (16), and 11.1% in the United States (17). There is limited information on the prevalence of these genes in Iran. Therefore, the current study aimed at evaluating the prevalence of new plasmid-mediated quinolone-resistance genes, *qnrA, qnrB,* and *qnrS*, among ESBL-positive and ESBL-negative *K. pneumoniae* isolates.

## Materials and Methods

**Specimen collection**

The current cross sectional study was conducted at Imam Reza Hospital in Mashhad, Iran. The study protocol was approved by the Ethics Committee of Mashhad University of Medical Sciences. One hundred and thirty isolates of *K. pneumoniae* were collected from May 2011 to July 2012. Clinical samples including urine, blood, wound culture, and cerebrospinal fluid (CSF) were processed in the current study.

**Antimicrobial susceptibility testing**

Antimicrobial susceptibility for ciprofloxacin, ceftazidime, cefotaxime, and cefpodoxime were assessed for the ESBL-producing *Escherichia coli* *(E. coli)* and non-ESBL-producing *E. coli,* by the standard disc diffusion method according to the Clinical and Laboratory Standards Institute (CLSI) guidelines (18). Isolated *E. coli* were cultured on Mueller-Hinton agar, and ciprofloxacin (5 µg), ceftazidime (30 µg), cefotaxime (30 µg), and cefpodoxime (10 µg) disks (Mast, UK) were placed on the inoculated plates and incubated at 35°C for 16 to 20 hours. Resistance to the studied antibiotics was assessed according to the growth inhibition zone sizes, compared with the CLSI breakpoints for *Enterobactericeae* (18).

**Phenotypic confirmatory test**

Phenotypic production of ESBL was determined by the combination disc method. According to CLSI guidelines, the bacteria were cultured on Mueller-Hinton agar, and ceftazidime (30 μg) versus ceftazidime/clavulanate (30/10 μg), cefotaxime (30 μg) versus cefotaxime/clavulanate (30/10 μg), and cefpodoxime (10μg) versus cefpodoxime/clavulanate (30/10 μg) disks (Mast diagnostics, UK) were placed on the inoculated plate by 20 to 30 mm distance from each other. After 18 to 24 hours of incubation at 37ºC, ESBL-producing organisms were detected by increase of the inhibition zone diameter by at least 5 mm around antibacterial agents in combination with clavulanic acid (18). The reference strain, *K. pneumoniae* ATCC 700603, was used as an ESBL-positive control.

**DNA Extraction**

Two to three colonies of bacteria were resuspended in a 500-µL of sterile distilled water. Suspensions were heated at 100°C for 15 minutes. Then, they were centrifuged at 3000 g for 10 minutes for the precipitation of cell debris. The supernatant was transferred to a new microtube and stored at -20°C (19, 20). 

**PCR detection of** ***qnr*** **genes**

The polymerase chain reaction (PCR) was performed by the specific primers for target genes (as shown in Table 1). Master mixes were prepared for each gene separately. Each contained 2 µL of 10X PCR buffer, 1.75 mM of MgCl_2_ 50 mM, 200 µM of each 10 mM dNTPs, 1 U of Taq DNA polymerase enzyme, 500 nM of each primers qnrB/A (10 µM) and 400 nM of each primers qnrS (10 µM) in the total volume of 20 µL. Two microliters of DNA was added into the reaction mixture to amplify each of the *qnr* genes. Amplification program for *qnrA* was 5 minutes at 94°C followed by 35 cycles of 30 seconds at 94°C, 45 seconds at 50°C, and 45 seconds at 72°C; and a final extension at 72°C for 5 minutes. The program for *qnrB* was 5 minutes at 94°C followed by 35 cycles of 30 seconds at 94°C, 45 seconds at 53°C, and 45 seconds at 72°C; and 5 minutes at 72°C as the final extension. For *qnrS*, it was 2 minutes at 94°C followed by 35 cycles of 45 seconds at 94°C, 45 seconds at 55°C, and 45 seconds at 72°C, and 10 minutes at 72°C as the final extension. Amplicons were run on 1.5% agarose gel and the final product was stained with Green Viewer (Pars Tous, Iran).

**Table 1:** Primers used for PCR to Detect *qnr* Genes

Gene	Primer	Amplified Fragment (bp)	Reference
*qnrA*	F:5`-ATTTCTCACGCCAGGATTTG-3` R: 5`-GATCGGCAAAGGTTAGGTCA-3`	516	(17)
*qnrB*	F: 5`-GATCGTGAAAGCCAGAAAGG-3` R: 5`-ACGATGCCTGGTAGTTGTCC-3`	469	(17)

PCR, polymerase chain reaction; bp, base pair

## Results

**Figure 1:**
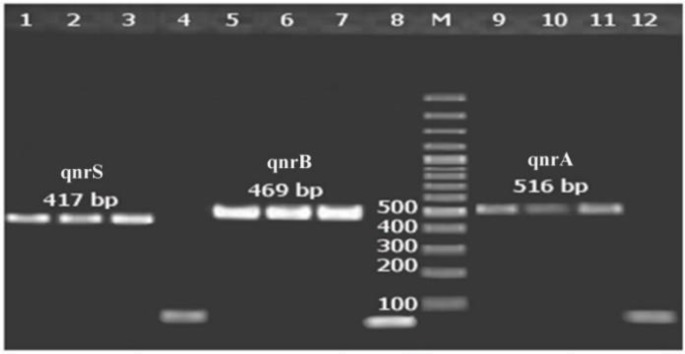
PCR results for qnrA, B, and S genes. Lane numbers 1, 2, and 3 show a 417-bp fragment of qnrS. Lane numbers 5, 6, and 7 show a 469-bp fragment of qnrB. Lane numbers 9, 10 and 11 show a 516-bp fragment of qnrA. Lane M represents a 100-bp DNA marker. Lanes 4, 8, and 12 are the negative controls

Seventy-nine (60.8%) of the 130 isolates were positive for *qnr* genes. The *qnrA, qnrS, *and *qnrB* were detected in 14 (10.8%), 20 (15.4%), and 27 (20.8%) isolates, respectively (as shown in Figure 1); 18 (13.8%) isolates possessed more than one *qnr* gene. The *qnrB* gene was the most common type of *qnr* genes; 56 (43%) isolates were capable of producing ESBL as evaluated by the confirmatory phenotypic test. The distribution of *qnr* genes in the clinical isolates of ESBL-KP and non-ESBL-KPs are shown in Table 2.

**Table 2:** The Distribution of *qnr* Genes in the Clinical Isolates of ESBL-*K. pneumoniae* and non-ESBL-*K. pneumoniae*

Number of qnr-genes positive results in clinical isolates of *K. pneumoniae*								
Isolate	*qnrA*	*qnrB*	*qnrS*	*qnrA, B*	*qnrA, S*	*qnrB, S*	*qnrA, B, S*	Total, N (%)
ESBL-positive(n=56)	3	16	8	3	2	5	2	39(%69.64)
ESBL-negative(n=74)	11	11	12	2	1	2	1	40(%54.05)

ESBL, extended-spectrum β-lactamase; *K. pneumoniae, Klebsiella pneumonia*

Totally, 88.6% (70 of 79) of the qnr-positive species were isolated from inpatients and 39 (49.4%) isolates were positive for ESBL (as shown in Table 3). Overall, out of 92% of the species isolated from inpatient samples, which were qnr-positive ESBL-positive *K. pneumoniae*, 64.2% were isolated from males and 35.8% from females. In the qnr-positive ESBL-producing *K. pneumoniae *isolates, 25% (10 of 39) possessed two *qnr* genes, and two isolates harbored three *qnr* genes. The results are shown in Table 3.

**Table 3.** Clinical Characteristics *qnr* Genotypes in the ESBL-Positive *K. pneumoniae* Isolates

Patient Characteristics				Nosocomial (n=36) 92.3%	Community Acquired (n=3) 7.7%	Total (n=39)
	Gender	Age (Mean=39.4)				
Demographic information	**Male** **(n=24)**		number	24(61.5)	-	24(61.5)
		Infant (0-12 months)	1			
		Child (1-5 year)	1			
		Young adult (18-40)	8			
		Middle age (40-60)	8			
		Elder (>60)	6			
	**Female** **(n=15)**	Infant (0-12 months)	2	12(30.7) 3(7.7) 15(38.4)		
		Child (1-5 year)	4			
		Young adult (18-40)	3			
		Middle age (40-60)	4			
		Elder (>60)	2			
Source of specimen		Urinary tract		18(46.2)	3(7.7)	21(53.9)
		Wound		5(12.8)	-	5(12.8)
		Blood		7(17.9)	-	7(17.9)
		Lower respiratory tract		4(10.3)	-	4(10.3)
		Eye		1(2.6)	-	1(2.6)
		Pus		1(2.6)	-	1(2.6)
qnr genes		B		18(46.2)	1(2.6)	19(48.8)
		A		1(2.6)	2(5.1)	3(7.7)
		S		6(15.4)	-	6(15.4)
		B/A		4(10.3)	-	4(10.3)
		B/S		4(10.3)	-	4(10.3)
		A/S		1(2.6)	-	1(2.6)
		B/A/S		2(5.1)	-	2(5.1)

ESBL, extended-spectrum β-lactamase; *K. pneumoniae, Klebsiella pneumoniae*

According to the data shown in Table 3, fifteen ESBL-positive species were isolated from subjects under five years old. Also, eight isolates were positive for *qnr* genes.

According to the results of the current study, 37.5% of ESBL-positive and 18.9% of ESBL-negative *K. pneumoniae* isolates were resistant to ciprofloxacin. Four of the ciprofloxacin-resistant ESBL^-^producing isolates and seven of ciprofloxacin-resistant non- ESBL**-**producing isolates had no *qnr* genes. Twenty-seven of 40 qnr-positive/ESBL-negative isolates, and 13 of 39 qnr*-*positive/ESBL-positive isolates were sensitive to ciprofloxacin. The *qnrB,* followed by *qnrS and qnrA,* was more prevalent in *K. pneumoniae* isolates. 

## Discussion

*K. pneumoniae* capable of producing ESBL is a concern both in the treatment and management of nosocomial infections. Plasmid-mediated antibiotic-resistance can easily be transferred between bacteria, which increase drug resistance in bacteria. Consequently, it may result in a high mortality rate among patients infected with such bacteria. In the current study, 37.5% (21 of 56) of ESBL-positive *K. pneumoniae* isolates were resistant to ciprofloxacin. The prevalence in the current study was higher than that of the study by Shahcheraghi (32%) (21), while it was lower than those of other studies by Wang et al. (42%), Robicsek et al. (69%), and another study by Wang et al. (73.9%) (6, 17, 22).

In a similar study conducted in Mashhad, Iran, the prevalence of ESBL-producing *E.coli* was high, especially in hospitalized patients (26). Furthermore, in a recent study performed in Mashhad, the most prevalent gene among all ESBL-producing *E.coli* isolates was *qnrA*, followed by *qnrB*, and *qnrS* (26).

In the current study, *qnr* genes were observed in 60.8% of *K. pneumoniae* isolates based on PCR findings. Compared to the results of the current study, the prevalence of *qnr* gene was higher in the studies carried out in China by Wang et al. (22.4%) and Jiang et al. (16.2%), in Korea by Kim et al. (40.5%), in Poland by Piekarska et al. (8.3%), and in Spain by Briales et al. (3.7%) (6, 7, 16, 23, 24).The current study results were lower than that of Richter et al. (68%) in Italy (25). In the current study, *qnrB* (32.31%), followed by *qnrS* (26.15%) and *qnrA* (19.23%), was more prevalent in *K. pneumoniae* isolates. In the studies by Wang et al., Jeong et al., Kim et al., and Piekarska et al. (6, 16, 23, 26), similar to the current study, *qnrB* was the most prevalent genotype. However, the most prevalent *qnr* gene in the studies carried out by Le et al., Jiang et al., and Wang et al. (7, 22, 27) was *qnrS*. Robicsek et al., showed that (17) *qnrA* was the most prevalent genotype. In the study by Wang et al. (6), similar to the current study, *qnrA* had the lowest prevalence. 

In the current study, 40 out of 74 ESBL-negative isolates were positive for any of the three *qnr* genes. The *qnrA* and *qnrS* were more prevalent in ESBL-negative isolates, compared with ESBL-positive isolates. The *qnr* genes were not found in 4 of 21 ciprofloxacin-resistant ESBL-positive isolates, and 7 of 14 ciprofloxacin-resistant ESBL-negative isolates. This may be due to chromosomal-mediated quinolone-resistance in the bacterial. Thirteen of 39 (33%) qnr-positive ESBL-positive isolates were sensitive to ciprofloxacin. Almost similar to the results of the current study, Saiful Anuar et al., found *qnr* genes in 7 (38.9%) sensitive isolates (15); however, these isolates were resistant to gentamicin or/and co- trimoxazole. Eighteen percent of ciprofloxacin-sensitive species isolated from wound specimens with mixed infection harbored *qnr* genes (11). 

In the current study, *qnr* genes were found in 8 out of 15 ESBL-positive *K. pneumoniae* species isolated from children. This result was consistent with that of Wang et al., which reported the rate of 11.9% (8 of 67) (22). Since quinolones are not recommended for children, acquisition of qnr-positive strains may occur via horizontal transmission by parents or via strains carrying transferable plasmids containing *bla* genes linked to *qnr* genes, which are acquired from hospital or other reservoirs (such as animal and agricultural products).

## Conclusion

In the current study, the prevalence of *qnr* among the clinical isolates of *K. pneumoniae* was 60.8% (79 out of 130). The current study indicated that among quinolone-resistant *K. pneumoniae* strains, ESBL-positive isolates were more prevalent that ESBL-negative ones. This could happen as a result of insertion in the same plasmid, which could in turn be easily transmitted to other microorganisms and increase the number of multidrug resistant isolates. High prevalence of quinolone-resistant genes among bacteria at Imam Reza Hospital of Mashhad is a major concern. Hence, antibiotics prescription pattern should be revised and the infection control measures should also be improved.

## References

[1] Coban AY, Nohut OK, Tanriverdi Cayci Y, Bayramoglu G, Pirincciler M, Cetinkaya E (2012). [Investigation of plasmid-mediated quinolone resistance determinants in enterobacteriaceae: a multicenter study]. Mikrobiyol Bul.

[2] Jacoby GA (2005). Mechanisms of resistance to quinolones. Clin Infect Dis.

[3] Luzzaro F (2008). [Fluoroquinolones and Gram-negative bacteria: antimicrobial activity and mechanisms of resistance]. Infez Med.

[4] Nordmann P (2006). [Emergence of plasmid-mediated resistance to quinolones in Enterobacteriaceae]. Pathol Biol (Paris).

[5] Martinez JL, Alonso A, Gomez-Gomez JM, Baquero F (1998). Quinolone resistance by mutations in chromosomal gyrase genes Just the tip of the iceberg?. J Antimicrob Chemother..

[6] Wang A, Yang Y, Lu Q, Wang Y, Chen Y, Deng L (2008). Occurrence of qnr-positive clinical isolates in Klebsiella pneumoniae producing ESBL or AmpC-type beta-lactamase from five pediatric hospitals in China. FEMS Microbiol Lett.

[7] Jiang Y, Zhou Z, Qian Y, Wei Z, Yu Y, Hu S (2008). Plasmid-mediated quinolone resistance determinants qnr and aac(6')-Ib-cr in extended-spectrum beta-lactamase-producing Escherichiacoli and Klebsiella pneumoniae in China. J Antimicrob Chemother.

[8] Nordmann P, Poirel L (2005). Emergence of plasmid-mediated resistance to quinolones in Enterobacteriaceae. J Antimicrob Chemother.

[9] Paterson DL, Mulazimoglu L, Casellas JM, Ko WC, Goossens H, Von Gottberg A (2000). Epidemiology of ciprofloxacin resistance and its relationship to extended-spectrum beta-lactamase production in Klebsiella pneumoniae isolates causing bacteremia. Clin Infect Dis.

[10] Zhang R, Wang XD, Cai JC, Zhou HW, Lv HX, Hu QF (2011). Outbreak of Klebsiella pneumoniae carbapenemase 2-producing K pneumoniae with high qnr prevalence in a Chinese hospital. J Med Microbiol.

[11] Martinez-Martinez L, Pascual A, Jacoby GA (1998). Quinolone resistance from a transferable plasmid. Lancet.

[12] Liao CH, Hsueh PR, Jacoby GA, Hooper DC (2013). Risk factors and clinical characteristics of patients with qnr-positive Klebsiella pneumoniae bacteraemia. J Antimicrob Chemother.

[13] Ruiz E, Rojo-Bezares B, Saenz Y, Olarte I, Esteban I, Rocha-Gracia R (2010). Outbreak caused by a multi-resistant Klebsiella pneumoniae strain of new sequence type ST341 carrying new genetic environments of aac(6')-Ib-cr and qnrS1 genes in a neonatal intensive care unit in Spain. Int J Med Microbiol.

[14] Strahilevitz J, Jacoby GA, Hooper DC, Robicsek A (2009). Plasmid-mediated quinolone resistance: a multifaceted threat. Clin Microbiol Rev.

[15] Saiful Anuar AS, Mohd Yusof MY, Tay ST (2013). Prevalence of plasmid-mediated qnr determinants and gyrase alteration in Klebsiella pneumoniae isolated from a university teaching hospital in Malaysia. Eur Rev Med Pharmacol Sci.

[16] Kim MH, Lee HJ, Park KS, Suh JT (2010). Molecular characteristics of extended spectrum beta-lactamases in Escherichia coli and Klebsiella pneumoniae and the prevalence of qnr in extended spectrum beta-lactamase isolates in a tertiary care hospital in Korea. Yonsei Med J.

[17] Robicsek A, Strahilevitz J, Sahm DF, Jacoby GA, Hooper DC (2006). qnr prevalence in ceftazidime-resistant Enterobacteriaceae isolates from the United States. Antimicrob Agents Chemother.

[18] d'Azevedo PA, Goncalves AL, Musskopf MI, Ramos CG, Dias CA (2004). Laboratory tests in the detection of extended spectrum beta-lactamase production: National Committee for Clinical Laboratory Standards (NCCLS) screening test, the E-test, the double disk confirmatory test, and cefoxitin susceptibility testing. Braz J Infect Dis.

[19] Izadi N, Naderi Nasab M, Harifi Mood E, Meshkat Z (2014). Prevalence of TEM and SHV Genes in Clinical Isolates of Klebsiella PneumoniaFrom Mashhad, North- East Iran. Iranian Journal of Pathology.

[20] Zaniani FR, Meshkat Z, Naderi Nasab M, Khaje-Karamadini M, Ghazvini K, Rezaee A (2012). The Prevalence of TEM and SHV Genes among Extended-Spectrum Beta-Lactamases Producing Escherichia Coli and Klebsiella Pneumoniae. Iran J Basic Med Sci.

[21] Shahcheraghi F, Moezi H, Feizabadi MM (2007). Distribution of TEM and SHV beta-lactamase genes among Klebsiella pneumoniae strains isolated from patients in Tehran. Med Sci Monit.

[22] Wang A, Yang Y, Lu Q, Wang Y, Chen Y, Deng L (2008). Presence of qnr gene in Escherichia coli and Klebsiella pneumoniae resistant to ciprofloxacin isolated from pediatric patients in China. BMC Infect Dis.

[23] Piekarska K, Rzeczkowska M, Zacharczuk K, Chrost A, Januszkiewicz A, Bareja E (2012). [Prevalence of qnr genes in clinical Enterobacteriaceae non-susceptible to fluoroquinolone in Poland]. Med Dosw Mikrobiol.

[24] Briales A, Rodriguez-Martinez JM, Velasco C, de Alba PD, Rodriguez-Bano J, Martinez-Martinez L (2012). Prevalence of plasmid-mediated quinolone resistance determinants qnr and aac(6')-Ib-cr in Escherichia coli and Klebsiella pneumoniae producing extended-spectrum beta-lactamases in Spain. Int J Antimicrob Agents.

[25] Richter SN, Frasson I, Bergo C, Manganelli R, Cavallaro A, Palu G (2010). Characterisation of qnr plasmid-mediated quinolone resistance in Enterobacteriaceae from Italy: association of the qnrB19 allele with the integron element ISCR1 in Escherichia coli. Int J Antimicrob Agents.

[26] Jeong HS, Bae IK, Shin JH, Jung HJ, Kim SH, Lee JY (2011). Prevalence of plasmid-mediated quinolone resistance and its association with extended-spectrum beta-lactamase and AmpC beta-lactamase in Enterobacteriaceae. Korean J Lab Med.

[27] Le TM, Baker S, Le TP, Cao TT, Tran TT, Nguyen VM (2009). High prevalence of plasmid-mediated quinolone resistance determinants in commensal members of the Enterobacteriaceae in Ho Chi Minh City, Vietnam. J Med Microbiol.

